# A cluster randomised controlled trial evaluating the effectiveness and cost-effectiveness of the daily mile on childhood obesity and wellbeing; the Birmingham daily mile protocol

**DOI:** 10.1186/s12889-017-5019-8

**Published:** 2018-01-11

**Authors:** Katie Breheny, Peymane Adab, Sandra Passmore, James Martin, Emma Lancashire, Karla Hemming, Emma Frew

**Affiliations:** 1 Health Economics Unit, Institute of Applied Health Research, University of Birmingham, B15 2TT, Birmingham, UK; 2 Institute of Applied Health Research, University of Birmingham, B15 2TT, Birmingham, UK; 3 Services for Education, Unit 3, Holt Court, Holt Street, Birmingham, B7 4AX UK

**Keywords:** Cluster randomised controlled trial, Childhood obesity prevention, Physical activity, Cost-effectiveness, School-based intervention, Wellbeing

## Abstract

**Background:**

Childhood obesity prevention is a public health priority. Children spend a large proportion of their waking time in school; therefore this is an appropriate setting to implement obesity prevention initiatives. Anecdotal reports suggest that implementing The Daily Mile in schools has had positive effects on childhood obesity, academic attainment and wellbeing. This trial aims to measure the effectiveness of The Daily Mile for improving health and wellbeing.

**Methods:**

This protocol describes a cluster randomised controlled trial (RCT) in 40 primary schools located in Birmingham, UK. Eligible participants are children in years 3 (aged 7–8) and 5 (aged 9–10). The study compares The Daily Mile (intervention) to usual practice (control) in relation to health and wellbeing. The Daily Mile intervention involves an additional 15 min of running or walking integrated into the school day, throughout a 12 month study period. The primary clinical outcome is body mass index (BMI) z-scores at 12 months following introduction of the intervention. The cost per Quality Adjusted Life Year (QALY) is the primary outcome of the economic evaluation. Secondary outcomes include wellbeing, physical fitness and teacher reported academic attainment.

**Discussion:**

This study is the first RCT investigating the clinical and cost-effectiveness of The Daily Mile. A range of outcomes will be measured to evaluate the broader wellbeing and academic benefits in addition to clinical outcomes typically measured in childhood obesity prevention trials. The intervention is simple and low-cost, therefore if the benefits are demonstrated it has enormous potential to influence future policy.

**Trial registration:**

ISRCTN: 12698269. Date protocol registered 27th October 2016.

**Electronic supplementary material:**

The online version of this article (10.1186/s12889-017-5019-8) contains supplementary material, which is available to authorized users.

## Background

Childhood obesity and overweight is a significant concern. In the 2015/2016 school year, 22.1% of children beginning primary school in England (reception, aged 4–5 years) were classified as overweight or obese. At primary school leaving age (year 6, aged 10–11 years) this figure was even higher, at 34.1% [1]. Not only does overweight at this age have immediate health implications such as increased risk of hypertension and insulin resistance [2], there is evidence that overweight children experience social rejection, discrimination and negative stereotyping [3]. The risk of these children becoming overweight adults is at least twice as high compared to normal weight children [4] and in the long-term, obesity and overweight are associated with premature death, increased risk of cardiovascular disease [5] and cancer [6].

Schools have been identified as effective settings to deliver interventions to prevent childhood obesity [7, 8]. The most recent Cochrane review [8] found a standardised mean change in Body Mass Index (BMI) of −0.14 (95% CI -0.21, −0.08) for interventions delivered in education settings alone. The effect increases when focusing on children aged between 6 and 12 years old only (−0.17, 95% CI -0.23 to −0.08), although caution is noted due to study heterogeneity and small sample sizes. A more recent review that focused only on interventions with a school-based component [7] found effectiveness of interventions delivered exclusively in schools had a mean difference in BMI z-score of −0.05 (95% CI -0.10, −0.01) or BMI -0.30 (95% CI -0.45, −0.15). The strength of evidence for school-based interventions was determined to be moderate.

Current physical activity guidelines recommend that children and young people between 5 and 18 years old should engage in at least 60 min of moderate to vigorous physical activity daily [9]. This is based upon evidence of the long-term benefits of physical activity, to include both physical and psychological well-being. The UK Government Childhood Obesity Strategy [10] recommends that of these 60 min, at least 30 min should be delivered in school time. Whilst active break times and physical education (PE) are opportunities for providing this volume of physical activity, issues with timetabling PE in a busy curriculum and ensuring play is active means achieving this daily target may be difficult. The Childhood Obesity Strategy [10] mentions The Daily Mile as an option to aid schools in delivering this volume of physical activity.

### The daily mile

The Daily Mile is an initiative developed and first implemented in a primary school in Stirling, Scotland in 2012. Initially designed to improve pupil’s fitness it is a simple and inclusive activity that increases children’s physical activity in school. In addition to improving physical health; further possible benefits include improved emotional wellbeing and academic achievement. The Daily Mile involves children doing an extra 15 min of activity by running or walking around a track within the school grounds. The 15 min reflects a distance of approximately one mile. Teachers can chose to do The Daily Mile at any time during the school day and in almost any weather, however it is not supposed to replace PE, break times or take place before or after school.

The Birmingham Daily Mile trial aims to conduct a robust evaluation of the effectiveness and cost-effectiveness of The Daily Mile in primary schools over 12 months. Despite anecdotal reports of its success in improving children’s fitness and wellbeing, as well as low rates of overweight in Stirling, there is currently no randomised controlled trial evidence supporting this. In addition, this study will assess the cost-effectiveness of the intervention. The Daily Mile may appear to incur no costs; however time and resources are incurred setting up and maintaining the intervention in the school day. The comparator is what the schools are routinely delivering to support health and wellbeing. This will enable a clear indication of the effect of introducing The Daily Mile to supplement existing initiatives.

### Trial aims and objectives

The aim of this pragmatic cluster RCT is to assess the clinical and cost-effectiveness of The Daily Mile in Birmingham primary schools for the purpose of improving health and wellbeing. The comparator intervention is usual practice (control arm), with schools receiving no additional physical activity intervention. Assessments of effectiveness will be conducted at 4 and 12 months following the initiation of the intervention. An economic evaluation will also be conducted to assess cost-effectiveness. The analysis will be conducted with and without implementation costs of the intervention. Qualitative methods will be used to explore the implementation of The Daily Mile and the measures used within the trial.

## Methods

### Trial design and overview

**Fig. 1:**
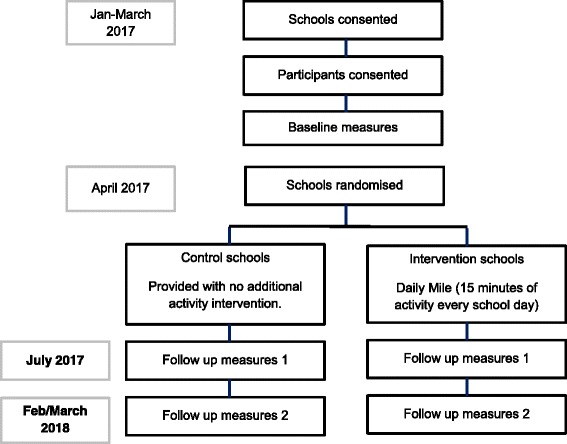
Study design of The Birmingham Daily Mile trial

The Birmingham Daily Mile study is a pragmatic cluster RCT conducted in the large, multi-ethnic city of Birmingham in the UK. Primary schools are the unit of randomisation, with data collected at the cluster (school) and within cluster (individual children) level. Schools are randomised to either the intervention (Daily Mile) or control (usual practice) arm. To evaluate the effect of The Daily Mile, several clinical outcomes and quality of life/wellbeing measures are being collected. These are described later. Baseline measures were undertaken on all eligible participants prior to school randomisation (Spring 2017). Follow up measures will be undertaken 4 months post introduction of the intervention and again after 12 months. The study design is summarised in Fig. 1. Intervention schools will be expected to maintain implementation of The Daily Mile throughout the 12 month study period.

### Study setting and participant eligibility

All Birmingham, UK schools with at least 20 pupils in school years 3 (aged 7–8 years) and 5 (aged 9–10 years) were eligible for participation in the Birmingham Daily Mile study. Initially eligible schools from an ethnically and socio-economically diverse part of the city (Northfield) were invited to participate and schools that expressed an interest in the trial were enrolled. Subsequent pragmatic invitation of eligible schools from a wider area was used to reach the recruitment target of 40 schools whilst ensuring the final sample included schools that varied in terms of ethnic make-up and levels of deprivation. The recruitment of forty schools will provide a sample of approximately 2000 children at follow-up.

#### Exclusion criteria

Pupils that had a disability preventing them running or walking for 15 min and those that were unable to have their height and/or weight measured at baseline were excluded.

### Study recruitment

#### School recruitment process

Schools were approached by email, summarising the study and inviting them to attend a briefing event where the study would be described in detail. If unable to attend the briefing they could obtain further information and discuss participation with the study coordinator at another opportunity. Follow-up communication was by email and telephone.

#### Recruitment of study participants

Pupils from one class in years 3 and 5 at participating schools were invited to take part in study measurements. Parents or caregivers received a letter describing the study and measurements it involved. If they agreed for their child to participate they provided consent by returning a signed consent form (Additional file 1). In addition, children were required to verbally assent to participating at the time of measurement. All children in schools allocated to the intervention arm will take part in The Daily Mile intervention. However, data collection is confined to those consented for study measurements.

### Trial intervention

The Daily Mile aims to increase children’s physical activity by at least 15 min every day. Schools map out a route or track in their school grounds. Once a day at a time chosen by the teacher, the whole class run or walk as far as they can around the track in 15 min. Participants are not required to change their clothes although in adverse weather, coats may be necessary. Teachers are encouraged to participate and to undertake The Daily Mile with their class in all weathers as long as it is safe to do so. Schools are directed to The Daily Mile website for additional resources [11]. They are able to incorporate The Daily Mile into the curriculum if they wish. For example participants could count and record laps for maths. However, the intervention should not be used to replace PE or break times. The intervention will run for 12 months during term time.

#### Comparator

Schools allocated to the control arm do not receive any intervention and continue with their usual practice in regard to health and wellbeing activities. Currently the amount of physical activity that primary schools should provide is not mandated, however at least 2 h a week is recommended by the Office for Standards in Education, Children’s Services and Skills (OFSTED) [12]. More recently, the Childhood Obesity Strategy states that schools should provide 30 min of moderate to vigorous activity daily [10]. Schools in this arm are requested not to commence any new health or physical activity initiatives during the 12 month intervention period.

### Method of random allocation and blinding

Randomisation was carried out after consent was taken for the school, and after baseline observations were made, to ensure allocation concealment. Randomisation was conducted using a constrained randomisation approach [13]. To this end, the school size, the average BMI Z-score, and proportion of pupils eligible for free school meals, were used in a balancing algorithm to generate a balance statistic. A random allocation was chosen by an independent statistician from those allocations that exhibited the greatest degree of balance on these three characteristics. Due to the nature of the intervention and comparator, clusters and participants will not be blinded to their intervention allocation. Blinding will be maintained for researchers who undertake follow up measurements.

### Outcome measures

The primary clinical effectiveness measure is the difference in BMI z-scores between the two study arms at 12 month follow-up. The primary outcome for the economic evaluation is the incremental cost per Quality Adjusted Life Year (QALY). The primary analysis is a cost-utility analysis however additional cost-effectiveness analysis using outcomes such as cost per unit of BMI z-score change or wellbeing will be conducted. Secondary outcomes include BMI z-score at 4 months, change in wellbeing, academic attainment, physical fitness and staff wellbeing (described in more detail under the data collection section below).

### Data collection

#### Clinical measurements

Participant’s height and weight are measured in school at baseline, 4 and 12 months, by trained researchers using a standard protocol. Measurements are taken without shoes on and while wearing light clothing. The Leicester Height Measure is used to measure standing height to the nearest millimetre. Height is measured twice, with a third observation taken if the values differ by greater than 4 mm. Weight (to the nearest 0.1 kg) and body fat percentage are measured using a Tanita bioimpedance monitor (Tanita SC-331S; Tanita Corporation., Tokyo, Japan). BMI z-scores are calculated using LMSgrowth software [14] which uses age and gender specific British 1990 growth data [15] to convert height and weight data to standard deviation scores using the LMS method [16].

Physical fitness is measured in school by school staff using the British Athletics Linear Track Test. Participants are encouraged to run as far as they can in two minutes on a pre-measured 50 m linear track. The distance achieved is recorded to the nearest 5 m and provides their endurance score.

#### Quality of life and wellbeing measures

Two quality of life/wellbeing questionnaires (Child Health Utility 9 Dimension (CHU 9D) and Middle Year Development Instrument (MDI)) are completed by the participants electronically in the classroom with teacher supervision. The CHU 9D is a generic health-related quality of life (HRQL) measure for children aged between 7 and 11 [17]. It is a self-report questionnaire requiring children to respond based on how they are feeling ‘today’. The questions address worry, sadness, pain, tiredness, annoyance, problems with school work, their daily routine and their ability to join in with activities. The CHU 9D was developed with the intention of being used as a preference based measure for use in paediatric economic evaluations. The MDI measures children’s social and emotional health and well-being in middle childhood (ages 6–12) [18]. This is a time when children experience important changes that establish their identity and impact their adolescent and adult development. The MDI is a self-report, multiple choice questionnaire asking children about their thoughts, feelings and experiences. Two index scores are produced: MDI Well-being Index and MDI Assets Index. Assets are quantities present in children’s lives that make a difference.

All class teachers are asked to complete a measure of wellbeing, the Warwick-Edinburgh Mental Well-being Scale (WEMWBS) [19]. This is a 14-item self-report questionnaire designed to measure mental wellbeing in adults and young people (aged 16 and above) over the last 2 weeks. It was developed in the UK and has been validated in a UK sample.

#### Sociodemographic data

Pupil’s date of birth, gender, ethnicity, postcode and free school meal eligibility are obtained from school records.

#### Academic attainment

Academic attainment of participants is reported by their teachers. Staff rate participants’ attainment in mathematics, writing and reading on a five point scale. The scale ranges from ‘below expected’ to ‘above expected’ and teachers are asked to rate them according to their Age Related Expectations.

#### School level data

Schools complete a questionnaire designed to explore their facilities, initiatives and general environment relating to food, physical activity and health. Questions relate to the school’s food policies and the promotion of healthy eating; the school’s physical activity policies and other healthy lifestyle initiatives and future plans. The questionnaire was adapted from a questionnaire used previously in the West Midlands ActiVe lifestyle and healthy Eating in School (WAVES) cluster randomised controlled trial [20]. Additional questions were also included to to enable assessment of the cost of the intervention and its implementation.

#### Process evaluation

Semi-structured interviews will be conducted with classroom staff and head teachers/deputy head teachers in participating schools. An estimated 30 interviews from 12 case study schools will be conducted, until saturation. A purposive sampling approach will be used, aiming to obtain a wide range of schools and staff’s views. School characteristics could include size of the school, OFSTED rating and level of deprivation. Participant characteristics may include socio-demographics, years of teaching experience and their job role. Qualitative data will be analysed using constant comparison methods. New data will be compared, initially to previous data and, as analysis progresses, to the properties of emerging categories.

The qualitative interviews will examine the barriers and facilitators to undertaking initiatives, such as The Daily Mile, aiming to improve health and wellbeing. How staff implemented The Daily Mile in intervention schools will be explored in addition to the long-term potential to include it in the school day. Perceived challenges and factors facilitating the initiative will also be addressed. For the purpose of the economic evaluation, further questions will investigate the opportunity costs of The Daily Mile and the appropriateness of the outcome measures used to demonstrate effectiveness in this setting.

### Justification of sample size

The sample size calculation was based on the primary outcome (BMI z-score) and allowed for the clustered nature of the trial and that the primary analysis would adjust for baseline value of BMI z-score. A follow-up sample of 2000 participants across 40 schools (50 participants per school) gives the study greater than 90% power to detect a 0.125 difference in BMI z-score between the Daily Mile and comparator arms – which is a difference used in previous childhood obesity prevent trials [20]. Based on unpublished data from other studies, we expect that the mean (SD) BMI z-score in the control arm will be 0.35 (1.3). The trial is powered with an intracluster correlation coefficient (ICC) of 0.04, and a correlation between baseline and follow-up observations of 0.9. However, the study is robust to any changes in these correlations, and will have greater than 90% power for values of the ICC between 0.001 and 0.07, and have greater than 80% power for values of the ICC between 0.07 and 0.10. The trial is also robust to changes in the correlation between baseline and follow-up observations, and will have greater than 80% power for values greater than 0.8. A 20% dropout rate would still provide greater than 80% power to detect the pre-specified difference in means. No allowance has been made for any variation in cluster sizes as this is expected to be minimal. The sample size calculation was conducted using the clustersampsi function in Stata [21].

### Data management

Electronic study data are stored on password protected systems and hard copies of data stored securely in locked premises. All data from paper record will be entered electronically by the study coordinator using original study forms returned by the participating schools.

### Planned statistical analysis

All statistical analysis will be conducted once the final set of measures (second follow-up) has been completed. Analyses will be by intention to treat and statistical significance will be considered at the 5% level (with 95% CIs reported). School and pupil level baseline characteristics will be presented by arm using appropriate summary measures (totals, percentages, means and standard deviations, medians and inter-quartile ranges). Characteristics will include age, ethnicity, deprivation, BMI z-score, physical fitness and wellbeing for individual participants. For schools; school size, ethnic mix and percentage eligible for free school meals will be reported.

As randomisation will be at the school (cluster) level, appropriate statistical methods to account for the clustering within schools will be used in the analysis. Analysis of outcomes will be for both 4 and 12-month follow-up measures. To evaluate effectiveness, a linear mixed model with follow-up outcome as the dependent variable and baseline values and treatment arm as the independent variables; with school included as a random effect. The primary analysis will also adjust for the variables used in the constrained randomisation (school size and proportion of pupils eligible for free school meals; and the average BMI Z-score of the school measured at baseline). We will allow for clustering at the school level and explore the possibility of allowing for an additional level of clustering at the year-group level. Secondary analysis will adjust for pre-specified pupil level factors which will include age, gender, baseline BMI z-score, ethnicity and deprivation from home postcode.

Outcome variables will be adjusted for the baseline values for the primary analysis. In addition, secondary analysis will adjust for pre-specified baseline school and child level covariates. These will include school level factors which were used in the randomisation (school size, average BMI z-score and % pupils eligible for free school meals) and pupil level factors (age, sex, baseline BMI z-score, ethnicity and deprivation from home postcode).

#### Planned subgroup analysis

Subgroup analysis will be conducted to assess the impact of the following characteristics on outcomes between the study arms: gender, year group, baseline BMI, deprivation level and ethnic mix of the school. The significance of a subgroup effect is assessed by an interaction test between the covariate and the treatment arm.

### Economic evaluation

The economic evaluation will take a public sector perspective and will comprise both a within trial analysis and a model based analysis that extrapolates beyond the study and enables the estimation of long-term cost-effectiveness. The Daily Mile will be compared to usual practice to estimate the incremental costs and benefits of implementing the intervention.

The within-trial analysis will assess the cost-effectiveness over the 12 month duration of the study. A micro-costing approach will be employed using data collected from the trial materials and qualitative interviews. Cost-utility analysis will be conducted with CHU 9D data used to estimate utilities for the calculation of QALYs. Cost-effectiveness analysis will use other effectiveness outcomes collected, including BMI and wellbeing. The results of both analyses will be presented as incremental cost-effectiveness ratios (ICERs). Sensitivity analysis will be conducted to test the uncertainty of the economic evaluation. Parameters such as cost will be varied to understand how they impact the results.

A model based analysis will also be conducted to consider the long-term cost-effectiveness of The Daily Mile for preventing obesity. Intermediate outcomes such as change in BMI will be linked to the consequences of childhood obesity in adulthood to predict the lifetime cost and outcomes of the intervention. Model inputs will be informed by literature reviews and sensitivity analysis will be conducted to assess the impact of assumptions, discount rate and model/parameter uncertainty. Costs and benefits will be discounted at a rate of 1.5%. This is the rate recommended by NICE for the evaluation of public health interventions [22].

## Discussion

Despite widespread uptake of The Daily Mile in the UK and its endorsement in UK policy documents, evidence for its success is currently only anecdotal. The Birmingham Daily Mile study will provide a robust evaluation of its impact on a variety of outcomes which include physical and, psychosocial health and academic attainment. Furthermore it will also evaluate its cost-effectiveness by including a within trial economic evaluation and decision analytic modelling. The Daily Mile is a simple and low-cost intervention which could provide a significant contribution to tackling childhood obesity. It therefore has great potential to impact health and education policy in the UK.

Childhood obesity is a complex issue with many contributing factors and no single solution [23]. The Daily Mile predominantly addresses one component, physical activity. There is no educational aspect to the intervention and no participation of family or caregivers outside of the school. Despite evidence suggesting that programmes involving both the home and school setting have most success in preventing childhood obesity [7], any contribution to reducing sedentary behaviour and perhaps encouraging additional activity outside of school is beneficial. Furthermore, by evaluating The Daily Mile in isolation and as it is currently being implemented will provide real-world evidence of its effect.

As a pragmatic study, a degree of variation in how well schools implement the study intervention is likely. Teachers are encouraged to use The Daily Mile as a tool for active learning or goal setting, for example, which may impact motivation or other contributors to implementation fidelity. Adherence to the recommended 15 min of activity daily may be variable and depend on other academic commitments or physical factors such as the weather. Teacher attitude may also affect whether children decide to continue doing activities similar to The Daily Mile outside of school such as on weekday evenings, at weekends or during school holidays. In addition, restricting the control arm schools from introducing new health initiatives for the duration of the study may also be ambitious. However, the large sample size, the participation of a diverse population and the inclusion of: cost-effectiveness; process evaluation and a variety of different outcomes are all strengths of this study which should provide a comprehensive assessment of The Daily Mile.

## Additional file

Additional file 1: Form for parents to consent to child measurements as part of trial. (DOCX 13 kb)

## Abbreviations

BMI: Body mass index
CHU 9D: Child Health Utility 9 Dimension
HRQL: Health-related quality of life
ICC: Intracluster correlation coefficient
ICER: Incremental cost-effectiveness ratio
MDI: Middle Years Development Index
OFSTED: Office for Standards in Education, Children’s Services and Skills
PE: Physical education
QALY: Quality adjusted life year
RCT: Randomised controlled trial
WAVES: West Midlands ActiVe lifestyle and healthy eating in school
WEMWBS: Warwick-Edinburgh Mental Well-being Scale
