# Supplementary material for: A cluster randomised controlled trial evaluating the effectiveness and cost-effectiveness of the daily mile on childhood obesity and wellbeing; the Birmingham daily mile protocol
Source: BMC Public Health. 2018 Jan 11;18:126. doi: 10.1186/s12889-017-5019-8 (PMC5765641; doi:10.1186/s12889-017-5019-8)
Supplement: Additional file 1: — Form for parents to consent to child measurements as part of trial. (DOCX 13 kb) [file 12889_2017_5019_MOESM1_ESM.docx]

# Additional file 1: Informed Consent Materials

**Run a Mile Study**

**PARENT CONSENT FORM**

I have read the information leaflet and:

(Please tick one option)

**DO GIVE CONSENT** **🞎**

**DO NOT GIVE CONSENT** **🞎**

for my child to take part in the Run a Mile Study measurements, and for [insert name] Primary School to provide information about my child’s date of birth, gender, ethnic group, school performance and postcode to members of the research team and to allow physical and emotional wellbeing to be assessed.

Child’s Name (Please Print)………………………………………………………………..

Class and School …………………………………………………………………………..

Parent/Guardian Name (Please Print)…………………………………………………….

Signature……………………………………………………………………………………..

Date……………………………………………………………………………………………

**PLEASE RETURN THIS CONSENT FORM TO YOUR CHILD’S SCHOOL BY [INSERT DATE]**
